# Novel identification of mixed infection of *Lactococcus garvieae* and *Cryptocaryon irritans* isolated from cultured *Trachinotus ovatus* in China

**DOI:** 10.1371/journal.pone.0301674

**Published:** 2024-07-23

**Authors:** Yucong Huang, Heng Sun, Jun Dong, Jianrong Zhang, Haoyu Wang, Lindi Yang, Yanping Li, Yifan Wang

**Affiliations:** 1 Guangdong Provincial Key Laboratory of Aquatic Animal Disease Control and Healthy culture & Key Laboratory of Control for Diseases of Aquatic Economic Animals of Guangdong Higher Education Institutes, Fisheries College of Guangdong Ocean University, Southern Marine Science and Engineering Guangdong Laboratory (Zhanjiang), Zhanjiang, GuangDong, China; 2 College of Veterinary Medicine, Southwest University, Chongqing, China; Tamil Nadu Dr J Jayalalithaa Fisheries University, INDIA

## Abstract

*Lactococcus garvieae* has recently been identified and listed as one of the causative agents of hyperacute hemorrhagic sepsis in fish. In intensive recirculating aquaculture systems where there are high fish densities and minimal water changes, not only will it be conducive to the growth of bacteria, but *Cryptocaryon irritans* as a marine protozoan fish parasite is also prone to appear. This study reports the disease status of *Trachinotus ovatus* in an aquaculture area in Yangjiang City, Guangdong Province. Through the diagnosis of clinical symptoms of the diseased fish, identification of specific primers, 16s rRNA sequences phylogenetic tree analysis, physiological and biochemical identification, and observation of histopathological sections, the result of the experiment is that the mass death of *T*. *ovatus* is caused by a mixture of *L*. *garvieae* and *C*. *irritants* infections. Subsequently, regression infection experiments were performed to verify Koch’s law. It was confirmed that the pathogen had strong virulence to *T*. *ovatus*. This is the first time that the co-infection of *L*. *garvieae* and *C*. *irritans* to *T*. *ovatus* was found in South China. The research results of this experiment have certain enlightenment significance for the epidemic trend of fish diseases in relevant sea areas.

## Introduction

Streptococcal infections of fish have become a severe problem worldwide with the intensification of aquaculture. *Lactococcus garvieae* which belongs to the *Streptococcus* family is a new opportunistic pathogen that affects a wide variety of fish species all over the world and causes serious economic losses as a result of high rates of mortality (up to 50%) and decreasing growth rates trends. This bacteria was initially isolated in a case study of bovine mastitis in the UK and was selected as a reference strain (ATCC 43921) for experiments with bacteria of the same species [1].

*L*. *garvieae* can infect a variety of seawater fish and freshwater fish including Japanese eel [2], Brazil Nile tilapia [3], pintado [3], olive flounder [4], kingfish [4], rainbow trout [5–8], catfish [9], freshwater prawn [10], bottlenose dolphin [11], and common octopus [12]. Physical evidence of *Lactococcosis* infection is the presence of hemorrhages in the periorbital and intraocular area, the base of fins, and the opercula. Internal organs are prone to hemorrhages and petechias at the surface. Furthermore, a rare cause of infective endocarditis is *L*. *garvieae*. An increasing number of human infections caused by *L*. *garvieae* has been reported in recent years. Handling and ingestion of raw fish are reported as a source or risk factor in the majority of clinical cases recorded [13], giving rise to the status of an emerging zoonotic pathogen.

*Cryptocaryon irritans* is also a major threat to tropical and subtropical marine bony fish [14]. In the meantime, it is easy to be infected with *C*. *irritants* in high-density marine aquaculture. There is no host specificity for *C*. *irritants* infections, and a high mortality rate is recorded in the alternate season of summer and autumn [15,16]. *C*. *irritants* have caused huge economic loss to the marine fish farming industry [17], and with a constant change in sea water temperature, there is an increase in the occurrence of infections resulting in high mortalities. *C*. *irritans* can severely impair the skin and gill function of a wide range of marine-farmed fish such as *T*. *ovatus*. More importantly, the probability of *C*. *irritans* leading to the death of *T*. *ovatus* is extremely high [18]. The life history of this ciliated protozoan includes 4 stages; larval stage, trophozoite stage, pre-cyst stage, and cyst stage [19]. Infected fish show anorexia, more mucus secretion, frequent rubbing of the skin on the side wall of the cage, among other abnormal behaviors. With the growth of *C*. *irritants*, dense white spots can be seen on the surface of the fish body, gills, eyes and an excessive mucus on the gills hindering the fish’s respiration, water circulation, and the discharge of harmful substances, which in turn cause mortality of the fish. Once *C*. *irritants* is present in aquaculture systems, it is very difficult to eradicate [20], and this is a major challenge being faced in the aquaculture industry.

Concerning mixed infection, several researchers have found that the growth and proliferation of *C*. *irritans* are intrinsically linked to bacterial pathogens such as *Vibrio harveyi* and *Staphylococcus aureus* through endosymbiotic relationships [21,22]. The proliferation of *C*. *irritans* is also influenced by environmental and physicochemical parameters [23]. The growth of *L*. *garvieae* is also strictly controlled by the environment, but the direct connection between the two is still unknown. *T*. *ovatus* which is a commercially important cultured marine fish, is widespread across China, Japan, and some other Asian countries [24–26]. The Guangdong Province is an important culture centre for tropical marine fish species in South China and *T*. *ovatus* production has expanded rapidly in this province in recent decades, but it has been seriously threatened by pathogens causing serious economic losses to farmers. Research and studies conducted found that the *T*. *ovatus* was mixed-infected by *L*. *garvieae* and *C*. *irritans* in Yangjiang, a ctiy in Guangdong province. However, few studies focusing on the mixed infections of *L*. *garvieae* and *C*. *irritans* in *T*. *ovatus* culture have been carried out. The purpose of this experiment was to discover, identify and report the situation of mixed infection. A brief study has been done to lay the foundation for further exploration of mixed infection.

## Materials and methods

### T. ovatus sampling

All cases recorded occurred at facilities practicing intensive farming. High mortalities of *T*. *ovatus* emerged in Yangjiang deep-sea aquaculture net cage of Guangdong Province from July to September. At the time of sampling, the water temperature in the mariculture area had exceeded 24°C. Five clinically diseased fish (body weight 500±25 g and body lenth 31.5±1.5cm, respectively) were collected from cages. All fish were transported alive in plastic bags with oxygen supplementation to the Laboratory.

### Macroscopic and histological analyses

The sampled *T*. *ovatus* were immediately euthanized with an overdose (190 mg/ L) of ethyl-m-aminobenzoate methane sulphonate (MS-222, Sigma) and dissected under aseptic conditions for bacterial and parasitological examination. Wet mounts of gill and mucus smears were examined macroscopically and microscopically for the detection of the presence of parasites.

For histological analyses, skin, brain, and kidney samples were taken from each fish; fixed in a solution containing 10% buffered formalin; dehydrated; and embedded in paraffin wax following standard protocol. Each tissue was sectioned at 5 μm and stained with hematoxylin and eosin. Sections were examined under a Zeiss^®^ microscope (Carl Zeiss AG, Jena, Germany).

### The isolation of bacteria

In a sterile environment, some spleen, kidney, and brain tissues were streaked onto Brain Heart Infusion (BHI) agar medium (QingDao Hope Bio-Technology Co., Ltd) and inoculated at 30°C for 48 h, after which the appearance of dominant and suspicious bacteria on the plate was noticed. Several single colonies were randomly selected and re-streak to obtain purification culture. The morphologically homogeneous colonies were collected and streaked onto new BHI agar to obtain pure cultures. All pure cultures were preserved at -80°C with 20% glycerol.

### Identification of bacteria and parasites

Total DNA from gills was extracted using TIANamp Marine Animals DNA Kit (TIANGEN Biotech) and was used for PCR amplification and gel electrophoresis. At the same time, natural seawater samples were collected from fish culture areas. The mixed microbial community samples were collected by filtering nature water samples through a 0.22 μm millipore filter membrane (Sangon Biotech, Shanghai, China), also used for DNA extraction and PCR detection. The specific primers used were Cryp-f and S15 (Table 1). And preliminary identification of isolated bacteria using specific primers ITSLg30F and ITSLg319R (Table 1) [27]. PCR reaction was performed with a final reaction volume of 50 μl containing 1 μl DNA template, forward and reverse primers 1.0 μL each, and 25 μl 2 ×Taq PCR superMix (Takara) in a thermocycler (BIO-RAD). PCR conditions were as follows: an initial denaturation of 94°C for 5 min, an annealing temperature of 55°C for 30s, an extension of 72°C for 40 s, and 35 cycles in total. The PCR products were analyzed by agarose gel electrophoresis. Following electrophoresis, gels were photographed under UV illumination. The PCR products were recovered with GeneJET PCR Purification kit (Thermo Scientific, USA), ligated with PMD18-T, and transformed into DH5α competent cells. Positive clones were selected and sent to Guangzhou Sangon Biotechnology Co., LTD for sequencing.

**Table 1:** Primers used for *C*. *irritans* and *L*. *garvieae* detection in *T*. *ovatus* from Yangjiang, Guangdong.

Primer	Sequence	Annealing temperature	Reference
Cryp-f	5′-CACTAGTTAGTGCGGGAAGT -3′	55°C	Xie Xiao et al. (2021)
S15	5′-TGAGAGAATTAATCATAATTTATAT-3′	55°C	Chen et al. (2008)
ITSLg30F	5’-ACTTTATTCAGTTTTGAGGGGTCT-3’	53°C	H T Dang et al. (2012)
ITSLg319R	5’-TTTAAAAGAATTCGCAGCTTTACA-3’	53°C	H T Dang et al. (2012)
27F	5’-AGAGTTTGATCCTGGCTCAG-3’	56°C	
1492R	5’-GGTTACCTTGTTACGACTT-3’	56°C	

### 16S rRNA sequencing and phylogenetic analysis

The universal primers 27F and 1492R (Table 1) were used to amplify the 16S rRNA genes of the isolated strain. The PCR reaction system was similar to that mentioned above. The reaction conditions were as follows: pre-denaturation at 94°C for 5 min, denaturation at 94°C for 30 s, renaturation at 57°C for 30 s, extension at 72°C for 90 s, and extension at 72°C for 10 min after 40 cycles. The procedure for sequencing PCR products was the same as in 3.4. The resulting 16S rRNA sequences were analyzed using the Basic Local Alignment Search Tool (BLAST). The isolated bacteria were named yx01, and the sequence was uploaded to NCBI. Also, four kinds of *Streptococcus* sequence and yx01 were used for phylogenetic and molecular evolutionary analyses of individual gene loci, and concatenated gene sequences were conducted using Molecular Evolutionary Genetics Analysis version 6 (MEGA 6). Phylogenetic trees were constructed from sequence alignments of concatenated sequence alignments using the Neighbor-Joining (NJ)[28]. method. Statistical support for the resulting nodes in each phylogenetic tree and all evolutionary analyses was done by the boot-strapping (BT) method with BT values set at 1000 replicates.

### Phenotypic and biochemical characterizations

The purified pathogenic strain was cultured on BHI solid medium at 28°C for 24 h, small, circular, raised, shiny, cream color, non-pigmented, colonies of yx01 were selected and inoculated in the microbiological reaction tube according to the physiological and biochemical instructions. The tests included Gram staining, catalase, motility, pH, temperature, and salinity, as well as the effects of ornithine, lysine, arginine, and other substances on the growth of bacteria results. Afterwards the culture was carried out at 28°C for 48 h. The results were observed, and the strain was preliminarily identified according to Bergey’s Manual of Systematic Bacteriology. Also, the results were compared with those of similar species.

### Koch’s postulates

The experimental fish (average body weight of 90.3± 8.5 g and average total body length of 18.8±1.1 cm, respectively) were healthy farmed *T*. *ovatus* free of pathogens. These fish were purchased from a fish farm with no history of the disease. Feeding was carried out twice a day with a commercial diet at approximately 5% of body weight and seawater was changed half everyday as well. The airstone was kept active and the water temperature was controlled at around 28°C over the experiment. The isolated strain yx01 was cultured overnight, then resuspended in PBS by centrifugation, and tenfold serial dilutions of the bacterial suspension were prepared. Fishes in the infection groups were respectively injected intraperitoneally with 0.1 mL of bacterial suspensions at doses of 1.12×10^8^, 1.12×10^7^, 1.12×10^6^, 1.12×10^5^ CFU/mL. Control fish were injected intraperitoneally with the same dose of sterile PBS. Clinical signs of disease and death status were observed and recorded daily for 14 days. The visceral organs of moribund fish were removed and subjected to bacterial isolation, purification and identification. After pure colonies were obtained, biochemical and PCR methods were used for identification.

## Institutional review board statement

All animal experiments were conducted strictly based on the recommendations in the ‘Guide for the Care and Use of Laboratory Animals’set by the National Institutesof Health. All fish experiments were approved by the Guide of the Animal Ethics Committee of Guangdong Provincial Key Laboratory of Aquatic Animal Disease Control and Healthy Culture., approval code: (20230110)02, approval date: 10 January 2023.

## Conclusion

### Clinical manifestations of diseased fish

**Fig 1:**
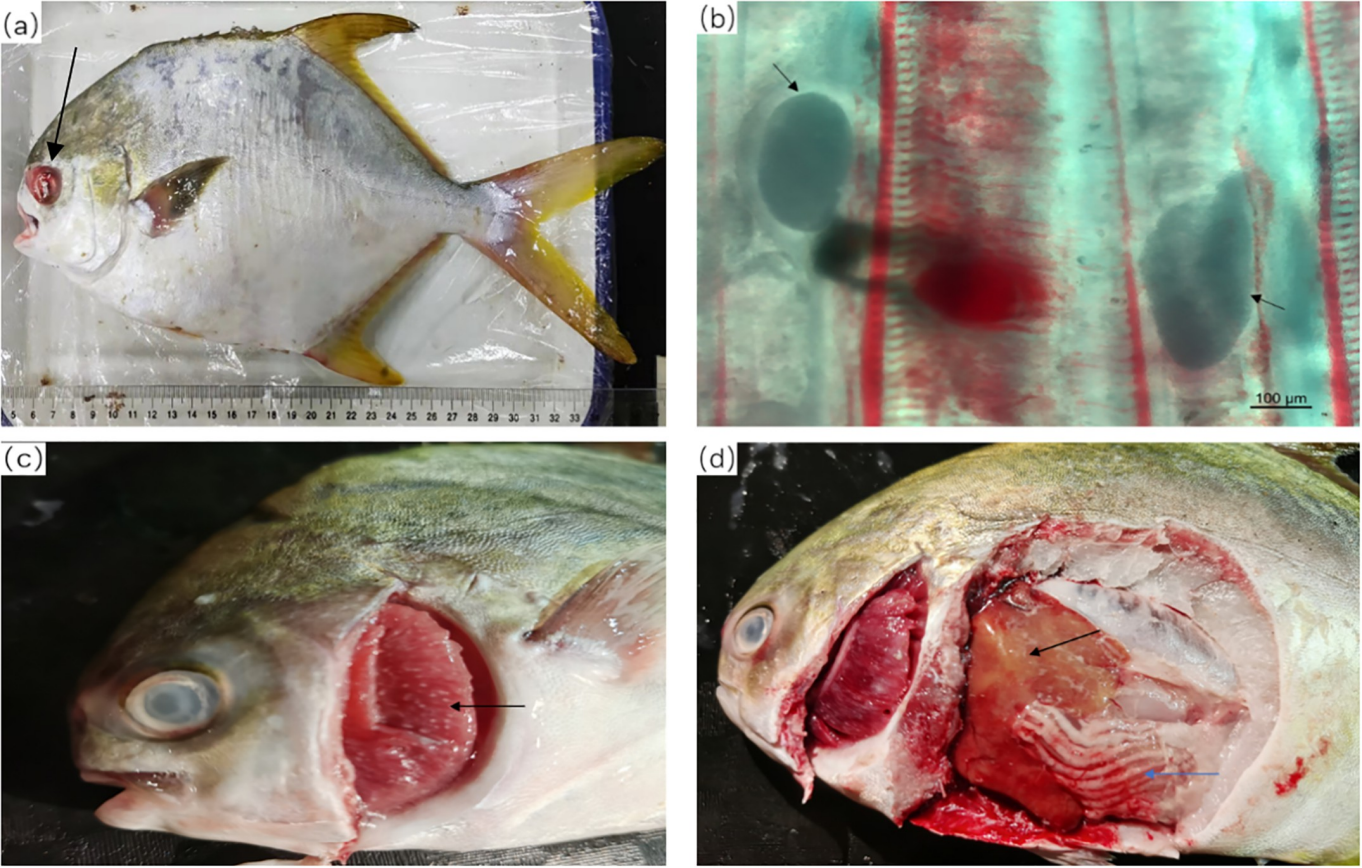
(a) Inflammation of the eyeball can be seen at the black arrow. (b) The existence of *C*. *irritans* on gill filaments (black arrow) can be observed. (c) It can be seen that there are many white spots on the gills (black arrow). (d) Black arrows point to liver tissue with uneven texture, and the blue arrow points to the inflamed intestine.

The sampled fish were selected based on their abnormal behaviors and macroscopic clinical signs, such as the appearance of a rapid and general anorexia, loss of orientation, erratic swimming, exophthalmia and inflammation of the eyeball, the presence of hemorrhages in the base of fins and the perianal region. There were many white spots on the body surface and gill filaments. The morphological characteristics of the parasites on the gills were preliminarily identified by microscope (Fig 1b).

After the autopsy, hepatomegaly, splenomegaly, intestinal inflammation (Fig 1d), and ascites were found.

### Observation and analysis of histopathological sections

**Fig 2:**
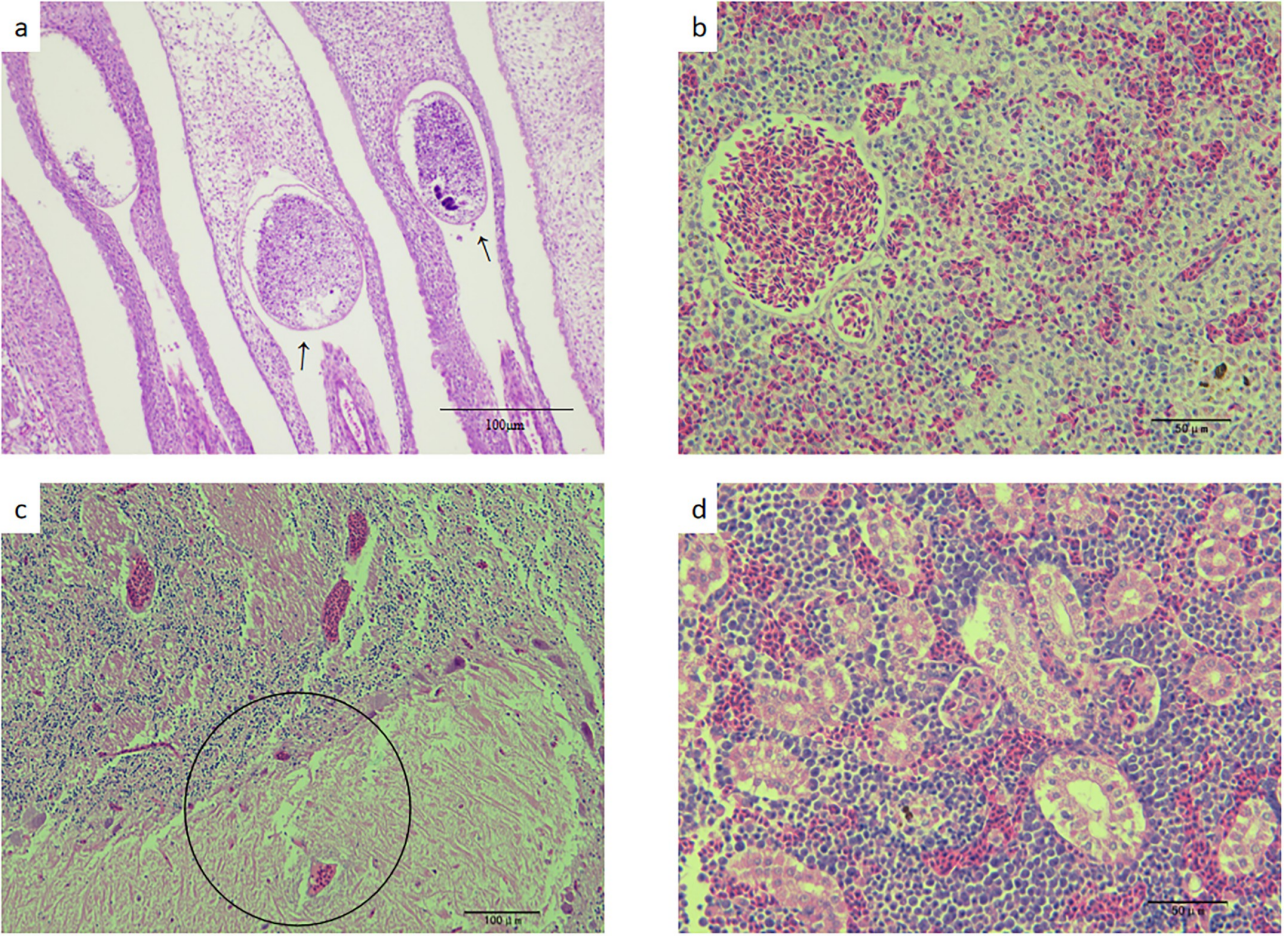
The pictures below are about the pathological manifestations of the sampled fish (a) Pathological section of gill filament, black arrow refers to *C*. *irritans*; (b) The congestion and hyperemia of the spleen are obvious; (c) Lymphocyte infiltration and congestion in brain tissue(circle);(d) Yellow arrows indicate lymphocytic infiltration and congestion, there is also renal tissue necrosis(black arrow) and there are also melanin-macrophage centers at the same time (blue arrow).

Histological lesions confirmed the presence of sepsis. Histopathologically speaking, the attachment of *C*. *irritans* was evident in the gill filaments (Fig 2a). On the spleen, hyperemia and congested were mainly observed (Fig 2b). Lymphocyte infiltration and congestion are common in brain tissues of diseased fish (Fig 2c). Hyperemia and necrosis of renal tissue, melanin macrophages in the center, and more lymphoid tissue hyperplasia were observed (Fig 2d).

### Detection of bacteria and parasites

**Fig 3:**
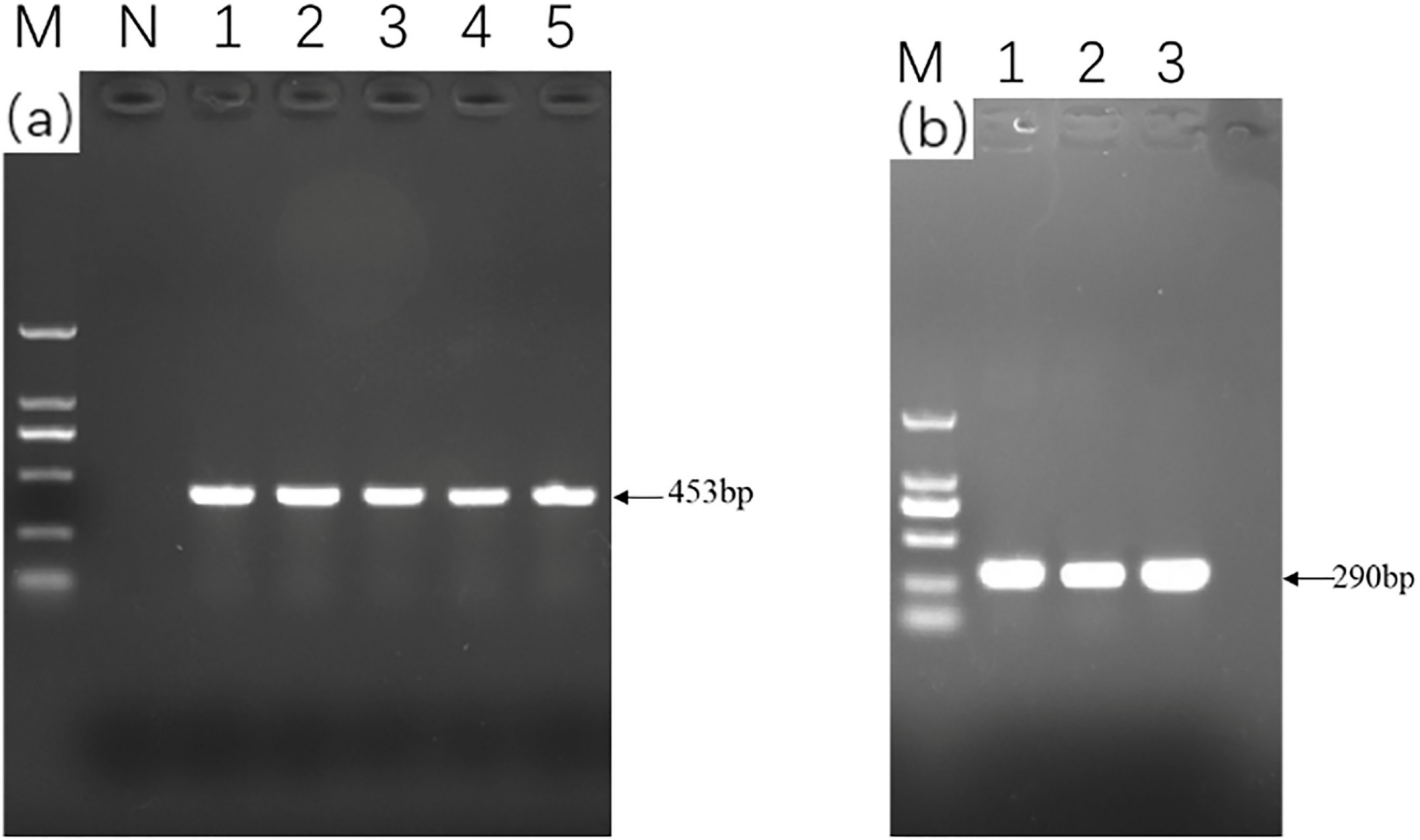
The results of gel electrophoresis are as follows. (a)Gel electrophoresis of sequence amplified by Cryp-F and S15 primer set. Line M is 2000bp DNA marker; Line N is a Natural seawater sample’s result; Line 1–5 are results of DNA from gills of diseased fish and fragment size is 453bp. (b) Line M is 2000bp DNA marker; Line 1–3 are the results of detection for diseased fish tissue DNA in PCR with ITSLg30F and ITSLg319R primers, fragment size is 290bp.

The presence of *L*. *garvieae* and *C*. *irritans* in tissues were detected using PCR with specific primers. As shown in Fig 3, After agarose gel electrophoresis, the result shows that there are *C*. *irritans* in the tissue and no results were detected in natural seawater samples (Fig 3a). It can be seen from Fig 3b that, the PCR fragments size is consistent with the expected size. At the same time, sequencing results of PCR products were put into the NCBI database for comparison, and it was found that *L*. *garvieae* and *C*. *irritans* were successfully isolated in this experiment.

### 16S rDNA sequencing and phylogenetic analysis

**Fig 4:**
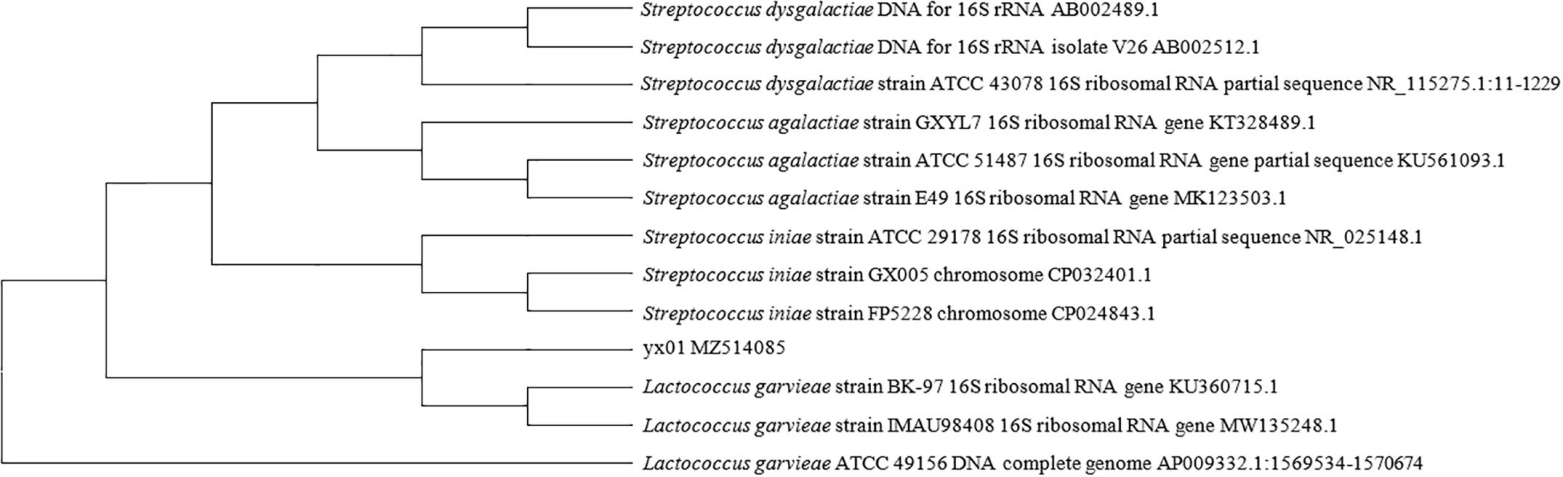
Phylogenetic analysis of strain yx01 and other Streptococci.

To determine the taxonomic position of yx01, 16S rDNA sequencing in combination with multi-locus sequencing was used for phylogenetic analysis. After sequencing of 16S rRNA amplification products by Sangon Biotech (Guangzhou) Co., Ltd, the sequences were uploaded to NCBI to obtain Genebank ID: MZ514085. Blast comparative analysis on NCBI found that yx01 was 98.9% similar to *L*. *garvieae*. As observed in Fig 4, the phylogenetic tree results show that strain yx01 has the highest similarity with *L*. *garvieae*.

### Bacteriological and biochemical analyses

Bacteria were isolated from the spleen and brain of diseased fish in a sterile environment. Purification and culture were done to obtain strain yx01 which is uniform in shape and size. Phenotypic and biochemical tests showed that the isolates identified were *L*. *garvieae* (Table 2). Growth of the isolated strain on BHI agar was tested at different temperatures and the results showed that it can grow at 4°C to 45°C (pH 5–9), but the growth rates were different. Bacteria yx01 was gram-positive, motile and negative for raffinose, xylose, sucrose, arabinose, urease, indole, H_2_S, citrate, lysine, and ornithine. Positive results were found for glucose, fructose, arginine dihydrolase, and mannitol. These results obtained were consistent with that of the reference strain ATCC43921 [29].

**Table 2:** Physiological and Biochemical Properties of yx01 and ATCC43921.

Items	yx01	ATCC43921 [29]
Gram	+	+
Motility	+	+
Growth in 4°C	+	+
In 10°C	+	+
In 25°C	+	+
In 35°C	+	+
In 45°C	+	+
pH4	--	--
pH5	+	+
pH8	+	+
pH9	+	+
Glucose	+	+
Raffinose	--	--
Xylose	--	--
Fructose	+	+
Sucrose	--	--
Arabinose	--	--
Urease	--	--
Indole	--	--
H_2_S	--	--
Arginine dihydrolase	+	+
mannitol	+	+
Citrate	--	--
Lysine	--	--
Ornithine	--	--

Note:”+” expressed as positive;”—” expressed as negative.

### Validation of the Koch hypothesis

After the strain yx01 was cultured, *T*. *ovatus* was injected intraperitoneally according to different concentrations (1.12×10^8^, 1.12×10^7,^ 1.12×10^6^, 1.12×10^5^ CFU/mL). The results showed that the fish from the high-concentration group died within 1–7 days after *T*. *ovatus* was infected. The fish from the low-concentration group died slowly, and the clinical signs of infected fish were similar to those of natural infection, while the control group maintained normal activities and no deaths occurred (Table 3).

**Table 3:** Mortality of *T*. *ovatus* challenged with yx01 by intraperitoneal injection.

Group	Concentration of bacterium (CFU/mL)	Dose (mL)	Number of experimental fish	Cumulative number of fish killed	mortality rate (%)
Experience group	1.12×10^8^	0.1	30	26	86.7
	1.12×10^7^		30	18	60.0
	1.12×10^6^		30	13	43.3
	1.12×10^5^		30	4	13.3
Control group	0.9%Physiological saline	0.1	30	0	0

In fishes infected with the yx01 strain with a concentration of 1.12×10^7^ CFU/mL or more were observed to be swimming slowly, and their food intake significantly reduced. Some infected fish had slight abdominal dropsy and signs of skin ulcers. In the experimentally infected groups, re-isolated strains obtained from spleen tissues from dying fishes were identical to strain yx01 in terms of morphology, physiological and biochemical characteristics, and 16S rRNA sequence. Also in this experiment, the LD_50_ of yx01 was 1.03×10^4^ CFU/g.

In contrast, no bacterial colonies were recorded for the control specimens. These findings indicate that this disease is propagated in *T*. *ovatus*, and strongly supports the high pathogenic potential of *L*. *garvieae* isolates from Yangjiang aquaculture.

## Discussion

In the present study, the bacteria isolated from naturally diseased *T*. *ovatus* was identified as *L*. *garvieae* and the parasite as *C*. *irritans*. In recent years, *L*. *garvieae* has been gradually discovered by researchers, but there are not many published cases, especially in the case where mixed infection of *L*. *garvieae* and *C*. *irritans* in *T*. *ovatus* is being studied. Thus, this experiment is the first report of its kind in the field of aquaculture.

The mortality of bony fish that has been naturally infected with *L*. *garvieae* has previously been reported to vary between 10%-50% [8], and the infection rate is dependent on the species, environmental stress, and water temperature [30]. A study conducted pointed out that, there is a high probability of disease and death of farmed fish when sea temperature exceeds 18°C [31]. In this study, the water temperature reached a temperature of 24°C, resulting in an extreme mortality rate recorded in the aquaculture area. Through the observation and analysis of clinical signs and pathological sections, it can be said that the large-scale death of *T*. *ovatus* was caused by mixed infections *L*. *garvieae* and *C*. *irritans*, which is similar to the clinical and macroscopic findings of other studies [32,33].

Regarding the Koch’s hypothesis and its verification, the water temperature was controlled at 28°C and a large number of experimental fish died within 3 days after intraperitoneal injection with a high concentration of bacteria. This confirms the high virulence of bacteria and also proved that the high mortality among *T*. *ovatus* in the aquaculture area was not only caused by *C*. *irritans*.

Simultaneously, the species of bacteria and parasites were preliminarily identified by specific primers in this experiment. The 16S sequence alignment on NCBI and the results of the phylogenetic tree further clarified the attribution of the bacterial types. Compared with the reference strain (ATCC43921) in the literature [29] from the perspective of physiology and biochemistry, the results were almost the same. Pathogenicity study in *T*. *ovatus* by experimental infection with *L*. *garvieae* yx01 validates Koch’s postulates, proving that the isolated strain is virulent.

In summary, the appearance of either *L*. *garvieae* or *C*. *irritans* will have a bad effect on the growth of *T*. *ovatus*. The most frightening event is when the two situations occur at the same time. The disease manifestations of cultured fish are more serious and the loss is immeasurable. However, there is currently no clear study confirming the sequence of mixed infections as well as the route [34]. *C*. *irritans* frequently appear during periods of large changes in sea temperature. *L*.*garvieae* also cause fish disease at high water temperatures. The real prevalence of the observed lesions is not complete. There is also limited literature related to this area of study, nonetheless, an increased frequency of reports in the last few years may indicate an increasing occurrence [29,35]. Although the results of this experiment expand the geographical distribution of *L*.*garvieae*, the prevention and treatment of mixed infections do require in-depth research and exploration, and they cannot be allowed to develop. In all, this study firstly isolated and identified mixed infections of *L*.*garvieae* and *C*. *irritans* outbreaks among *T*. *ovatus* cultured in Yangjiang aquaculture in Guangdong Province. This study also lays the foundation for the follow-up research on the mixed infection of *T*. *ovatus*.

## Supporting information

S1 File (XLSX)
